# Editorial: Carbapenemase-Producing Organisms as Leading Cause of Hospital Infections

**DOI:** 10.3389/fmed.2021.775021

**Published:** 2021-10-25

**Authors:** Spyros Pournaras, Raffaele Zarrilli, Paul G. Higgins, Constantinos Tsioutis

**Affiliations:** ^1^Laboratory of Clinical Microbiology, School of Medicine, Attikon University Hospital, National and Kapodistrian University of Athens, Athens, Greece; ^2^Department of Microbiology, School of Medicine, National and Kapodistrian University of Athens, Athens, Greece; ^3^Department of Public Health, University of Naples Federico II, Naples, Italy; ^4^Institute for Medical Microbiology, Immunology and Hygiene, University of Cologne, Cologne, Germany; ^5^School of Medicine, European University Cyprus, Nicosia, Cyprus

**Keywords:** carbapenem-resistance, metallo-β-lactamase, serine carbapenemase, healthcare-associated infection, infection control

Carbapenemase-producing Gram-negative organisms (CPO) currently constitute a severe public health problem. Among them, the most critical threat is posed by carbapenemase-producing *Enterobacteriacae* (CPE), carbapenem-resistant (CR) *Acinetobacter baumannii* (CRAB), and *Pseudomonas aeruginosa* (CRPA). Infections by these organisms are increasingly occurring worldwide, are associated with adverse patient outcomes and cause a significant burden on healthcare systems. These pathogens have the ability to spread rapidly among patients due to their ability to survive and propagate in the hospital environment, and antimicrobial resistance is further spread due to the mobile genetic elements carrying the responsible genetic loci. CPO are by definition resistant to multiple antibiotic classes, resulting in limited therapeutic options and difficult-to-treat infections, with high morbidity and mortality rates. In addition, prompt detection of these organisms is paramount for implementation of appropriate infection control measures and early management.

It is therefore evident that infections by CPO entail challenges in their detection, control, and management. Their global health implications are reflected in the increasingly recorded numbers of published reports, studies, and recommendations, emphasizing the urgent need for optimization of diagnostics and therapeutics, as well as for establishment of targeted and data-driven prevention and control policies.

This research topic intended to present to the readers important peer reviewed articles containing new knowledge on every aspect of CPO and their respective infections. It overall harbors 24 manuscripts, including original research, methods, and review articles addressing these subjects. The 173 authors involved in this topic mainly focused on the epidemiology, diagnostics, clinical characteristics, and therapeutics of CPO. A brief overview of the scientific content of the Topic is shown in this Editorial.

Several of the published manuscripts analyzed the molecular epidemiology of CPO. Carrasco et al. reported on the polymyxin resistance of an XDR ST1 carbapenem-resistant *A. baumannii* outbreak clone in a Brazilian teaching Hospital. They concluded that the emergence of polymyxin resistance in this high-risk global clone spreading in their hospital was due to mutations in the chromosome of the carbapenem-resistant ST1 isolates. Al-Hassan et al. analyzed the molecular epidemiology of 42 carbapenem-resistant *A. baumannii* from four hospitals in Khartoum State, Sudan. They found a predominance (88%) of international clone (IC) 2 with OXA-23, and some with NDM-1 carbapenemase. Isolates belonging to IC1, IC5, and IC9 were also identified. Solgi et al. reported the occurrence of OXA-48 and NDM-1 producing *Enterobacterales* species at a University hospital in Tehran, Iran, between 2015 and 2016. Two separate outbreaks of NDM-1-producing ST147 and OXA-48-producing ST893 *Klebsiella pneumoniae* and one outbreak of OXA-48-producing *Serratia marcescens* were observed. The *bla*_OXA−48_ gene was located on an IncL/M conjugative plasmid, while the *bla*_NDM−1_ gene was located on both IncFII ~86 to ~140-kb and IncA/C conjugative plasmids. Chudejova et al. analyzed whole genome sequences of four *Enterobacterales* isolates co-producing NDM- and OXA-48-like carbapenemases recovered from Czech hospitals, including three *K. pneumoniae* assigned to “high risk” clones ST147, ST11, and ST15 and one *Escherichia coli* assigned to ST167. All four isolates co-produced OXA-48- and NDM-type carbapenemases, in different combinations (*K. pneumoniae*: *bla*_NDM−5_ and *bla*_OXA−181_; *bla*_NDM−1_ and *bla*_OXA−181_; *bla*_NDM−1_ and *bla*_OXA−244_; *E. coli*: *bla*_NDM−5_ and *bla*_OXA−244_). The *bla*_OXA−244_ was found on a plasmid in one *K. pneumoniae* isolate, while the *bla*_OXA−244_ was localized in the chromosomal contig of the *E. coli* isolate. The *bla*_OXA−181_ was identified in two distinct plasmids. Also, the *bla*_NDM−1_ and the *bla*_NDM−5_ were found in four distinct plasmids of the respective isolates. Liu et al. studied the molecular characteristics of 12 *bla*_*IMP*__−*4*_-producing *Enterobacterales* from Henan province of China and their *bla*_*IMP*__−*4*_-carrying plasmids. N-type plasmids were the predominant plasmids carrying *bla*_*IMP*__−4_ among the collected *Enterobacterales* (8/12, 66.7%), were more stable than the other types of plasmids and were transferrable in three cases. Complete sequence analysis of a representative N type (pIMP-ECL14–57) revealed that it was nearly identical to pIMP-FJ1503, an N-type *bla*_*IMP*__−4_-carrying epidemic plasmid in a *C. freundii* strain. Okanda et al. examined 43 carbepenemase-producing *K. pneumoniae* isolates in the intensive care unit of a 1,900-bed hospital in Bangladesh, among which most were harboring *bla*_*NDM*−__1_ (23, 53%), *bla*_*NDM*−__5_ (6, 14%), and *bla*_*OXA*−__181_ (5, 12%), whereas 23% classified as extensively-drug resistant and 14% were classified as pandrug-resistant. A study in 80 transplant patients colonized or infected with carbapenemase-producing *K. pneumoniae* in Porto Alegre, Brazil by Raro et al. detected intrahospital and interhospital spread of epidemic clones ST11/KPC-2, ST16/KPC-2, ST15/NDM-1, and ST437/KPC-2, accounting for an overall mortality of 21.3%. Pan et al. investigated 50 carbapenem-resistant *Klebsiella aerogenes* from children in a pediatric hospital. Carbapenem resistance was mediated by NDM-5 in 45 of the isolates, and conjugation experiments followed by plasmid typing showed that the majority of plasmids carrying *bla*_NDM−5_ were IncX3. All the isolates belonged to ST-4, and ERIC-PCR suggested 13 clusters, with one containing 17 isolates. Molecular epidemiology of *Pseudomonas aeruginosa* was investigated by Rada et al. who described a wide variety of isolates harboring VIM-2 and KPC-2 from two hospitals, within and outside of Medillin, Colombia. Out of 46 isolates, 11 had *bla*_VIM−2_, 11 had *bla*_KPC−2_, and one had both genes. *bla*_VIM−2_ was associated with class 1 integrons, while *bla*_KPC−2_ was plasmid-encoded and contained within a Tn*4401*b transposon. Rep-PCR was used to initially type the isolates, and 16 were further investigated by WGS which revealed that most of the isolates were unrelated and assigned the 16 isolates to 9 different STs.

Analysis of genomic characteristics of CRE isolates, including mobile genes or new gene variants, was also reported by a series of manuscripts in this research topic. Mobile carbapenemase genes in *Pseudomonas aeruginosa* were reviewed by Jeong and Jeong. This review illustrated epidemiologically the carbapenem resistance in *P. aeruginosa*, including the resistance rates worldwide and the carbapenemase-encoding genes, the mobile genetic elements responsible for the horizontal dissemination of the drug resistance determinants and the modular mobile elements including carbapenemase-encoding genes (*P. aeruginosa* resistance islands). Yoon et al. identified a novel KPC variant, KPC-55, which exhibited improved meropenem-hydrolyzing activity, in *K. pneumoniae* of ST307. Compared to a KPC-2-producing isogenic strain, the KPC-55-producing strain exhibited a lower level of resistance to most β-lactam drugs tested, however, the KPC-55 enzyme hydrolyzed aztreonam and meropenem at an increased efficiency compared to the catalytic activity of KPC-2. Chen, Zhou. et al. reported whole genome sequence of NDM-7-producing *K. pneumoniae* strain HZW25 isolate from China and identified an IncX3 *bla*_NDM−7_-carrying conjugative plasmid, which could be horizontally transferred successfully. The dissemination of such NDM-producing *K. pneumoniae* would be troublesome during treatment using ceftazidime-avibactam. Chen, Lin et al. characterized a new transposon, Tn6696, on a *bla*_NDM−1_-carrying plasmid from multidrug-resistant *Enterobacter cloacae* ssp. *dissolvens* CBG15936 from China, providing another perspective regarding the potential for *bla*_NDM−1_ to undergo horizontal transfer among drug-resistant bacteria. In another study from China, Yao et al. described in *K. pneumoniae* an IncFII_k_ plasmid that harbored multiple antibiotic resistance determinants including *bla*_IMP−26_ and a *tet*(A) variant. The plasmid was found to confer resistance to multiple antimicrobials and raised the MIC of tigecyline 8-fold in the transformant. In another study, an IMP variant, IMP-8, was described by Guo et al. in a rarer organism, *Comamonas thiooxydans*. The organism was isolated from a mid-section urine specimen. *bla*_IMP−8_ was encoded in an integron, In 655, that is similar to that described in a *K. pneumoniae* isolate, but differed by the presence of two recombinases flanking the integron. The isolate was susceptible to imipenem and intermediate to meropenem, but exhibited an MDR phenotype that included cephalosporins, fluoroquinolones, and aminoglycosides. Lastly, a case report from China (Chen, Lin et al.) detected a new transposon, Tn6696, on a *bla*_NDM−1_-carrying plasmid in an *E. cloacae* ssp. *dissolvens* isolate, providing evidence for the potential of *bla*_NDM−1_ to undergo horizontal transfer.

Studies of this Topic that evaluated treatment options against carbapenemase-producing organisms include that of Cui, Shan et al. who found reduced susceptibility to ceftazidime-avibactam in 12 (3.5%) of 347 retrospectively tested KPC-producing *Klebsiella pneumoniae* isolated in Chinese patients, without previous exposure to ceftazidime-avibactam. Interestingly, these 12 isolates were from geographically distinct areas and all belonged to ST11. Again, in China, Qu et al. performed a checkerboard assay with Mueller-Hinton broth to study the *in vitro* effects of various antimicrobial combinations against 89 CRAB isolates (95.35% were *bla*_OXA−23_). Highest synergistic effects were recorded in sulbactam-based combinations with polymyxin B (82.35%) and tigecycline (73.91%). Interestingly, resistance mechanisms were unrelated to clinical outcome. A study from Spain (Cebrero-Cangueiro et al.) evaluated the efficacy of fosfomycin and its combination with aminoglycosides in an experimental sepsis model by carbapenemase-producing *Klebsiella pneumoniae* clinical strains. They observed a dissimilar efficacy of fosfomycin plus aminoglycosides in treating this severe experimental infection, when caused by different CPO and suggested that fosfomycin plus amikacin or gentamicin may be useful to treat infections by OXA-48 plus CTX-M-15 or KPC-3 producer strains, respectively.

Furthermore, in the research topic, an extensive review about carbapenem-resistant bacteria in patients with hematologic malignancies by Lalaoui et al. provided insights on the clinical and microbiological characteristics of these infections and proposed a literature-based algorithm for risk assessment and empirical management.

This research topic also presented studies reporting the diagnostics of CPO or carbapenem resistance phenotypes. A study from China (Zhang et al.) evaluated the performance of the BD Phoenix NMIC413 AST panel on a large collection of 195 CREs and 108 CSE isolates and showed very satisfactory concordance with BMD used as reference. Cui, Jia et al., reported a modification of the carbapenem inactivation method, using *Bacillus stearothermophilus* as an indicator strain, resulting in a rapid carbapenemase phenotype detection, requiring only 4 h of total work time and exhibiting high sensitivity, specificity and accuracy. Pfaendler et al. evaluated the phenotypic CarbaLux® test for routine diagnostics in the medical laboratory; they reported that this novel fluorescence method allowed simple and safe handling, reliable readings, and documentation of carbapenem resistance phenotypes. Bonnin et al. reviewed the genetic diversity, biochemical properties, and detection methods of minor carbapenemases in *Enterobacterales*. They correspond to class A (SME-, Nmc-A/IMI-, SFC-, GES-, BIC-like…), class B (GIM, TMB, LMB…), class C (CMY-10 and ACT-28), and class D (OXA-372) less frequently detected carbapenemases. The review of phenotypic and molecular methods for detection of minor carbapenemases and the use of β-lactamase inhibitors against minor carbapenemase-producing Enterobacterales was particularly of interest. Finally, Moreno-Morales et al. evaluated the efficacy of a loop-mediated isothermal amplification (LAMP) assay to detect the presence of carbapenemases in *Acinetobacter* spp. directly from bronchoalveolar lavage (BAL) samples spiked with 22 *Acinetobacter* spp. strains producing OXA-23, OXA-40, OXA-58, NDM, and IMP carbapenemases. The reported molecular diagnostics kit had enough sensitivity for the detection of carbapenemase-producing *Acinetobacter* in clinical BAL samples with the limit of sensitivity of 10^3^ CFU/ml with a maximum hands-on time of 15 min per sample and 30 min run time (~45 min total).

## Author Contributions

All authors listed have made a substantial, direct and intellectual contribution to the work, and approved it for publication.

## Conflict of Interest

The authors declare that the research was conducted in the absence of any commercial or financial relationships that could be construed as a potential conflict of interest.

## Publisher's Note

All claims expressed in this article are solely those of the authors and do not necessarily represent those of their affiliated organizations, or those of the publisher, the editors and the reviewers. Any product that may be evaluated in this article, or claim that may be made by its manufacturer, is not guaranteed or endorsed by the publisher.

